# The Development of Fat Embolism Syndrome (FES) and Multiple Small Pulmonary Emboli Following Open Reduction Internal Fixation (ORIF) of a Left Femur Fracture: A Case Report

**DOI:** 10.7759/cureus.45551

**Published:** 2023-09-19

**Authors:** Aaron Hacker, Dylan S Irvine, Scott MacDougal, Imani Thornton

**Affiliations:** 1 Anesthesiology, HCA Florida Westside Hospital, Plantation, USA; 2 Medicine, Nova Southeastern University Dr. Kiran C. Patel College of Osteopathic Medicine, Davie, USA; 3 Anesthesiology and Critical Care, HCA Florida Westside Hospital, Plantation, USA

**Keywords:** fat embolism syndrome, orthopedic procedures, vasopressor, distal femur fracture, pulmonary emboli

## Abstract

Fat embolism syndrome (FES) is a rare but potentially life-threatening complication that can occur following orthopedic procedures, such as long bone fracture repairs. FES is caused by the release of fat globules into the bloodstream, leading to the obstruction of blood vessels and subsequent tissue damage. Pulmonary embolism (PE), a condition in which a blood clot travels to the lungs, is another potential complication of orthopedic procedures due to the mobilization of blood clots during surgery. We report the case of a 56-year-old female who presented to the emergency department with a left femur fracture following a mechanical fall and underwent open reduction internal fixation (ORIF) surgery for the fracture. The procedure was complicated by the development of FES and multiple small pulmonary emboli. The patient was managed postoperatively in the ICU, requiring support with multiple vasopressors and mechanical ventilation. She remained in the ICU for three days postoperatively and was discharged on postoperative day six to an inpatient rehabilitation facility.

## Introduction

Fat embolism syndrome (FES), the systemic manifestation of fat emboli within the microcirculation, is a rare but potentially fatal complication of orthopedic injuries, such as femur fractures [1]. The pathophysiology of FES involves the release of fat globules into the bloodstream, which activates the complement system and results in leukocyte aggregation [1-2]. This can result in obstruction of blood vessels and subsequent tissue damage [1-2]. The incidence of FES has been reported in 0.5-11% of patients who experience long bone fractures [3]. FES can be fatal in approximately 1-10% of cases, although the incidence of severe complications has decreased, possibly due to preventative measures and improved intraoperative monitoring for patients at increased risk [1]. Measures that have been shown to decrease the likelihood and complications from FES include early fixation of fractures rather than delayed fixation, and modifications of reamer systems such as slim shafts, sharp tips, and enlarged flutes, which reduce intramedullary pressure peaks and the embolic load [1]. Enhanced observance of patient oxygenation status, hemodynamics, and cutaneous changes in those with higher-risk fractures, such as pathologic and bilateral fractures versus acute isolated fractures, are certain intraoperative monitoring methods that can also reduce FES incidence and improve outcomes [1].

The highest risk for morbidity and mortality in FES occurs in the setting of pre-existing respiratory compromise or cardiovascular complications [1,3]. Delay in the identification and treatment of FES leads to increased morbidity and mortality. FES can be diagnosed clinically based on the following triad of symptoms: respiratory distress, neurological dysfunction, and a petechial rash, or diagnostic criteria such as those proposed by Gurd and Wilson. There is no standard treatment protocol for FES [1,3-4]. The treatment is largely focused on supportive measures and patient stabilization that may include the administration of medications or other life-supporting measures to improve oxygen delivery to tissues, prevent further injury, and manage comorbidities [1]. The approach to management is influenced by the degree of severity and setting in which FES is identified [1-2]. Pulmonary embolism (PE) is another possible complication of operative procedures, in which a blood clot travels to the lungs and causes respiratory distress [5]. The treatment typically involves anticoagulant therapy, such as heparin or warfarin, to prevent further clot formation and dissolve existing clots [5].

## Case presentation

The patient was a 56-year-old female who presented to the emergency department with left lower extremity pain following a mechanical fall. She reported falling at home immediately prior to the arrival. She had experienced immediate left knee pain, swelling, and inability to ambulate or bear weight following the event. Upon physical exam, the patient was 162.6 cm in height and weighed 61 kg, with a body mass index (BMI) of 23.1 kg/m^2^. The left leg was observed to be short and externally rotated with tenderness to palpation over the proximal femur and groin. There were no gross deformities or open wounds of the left hip, and no bony crepitus or ligamentous laxity over the knee, ankle, or proximal tibia or fibula. Sensation was intact to light touch. Pedal pulses were 2+ and symmetric bilaterally. Pre-anesthesia vital signs included a blood pressure of 123/72 mmHg, heart rate of 61 beats per minute, respiratory rate of 16 breaths per minute, oxygen saturation of 96% on room air, and oral temperature of 97.5 °F.

**Figure 1:**
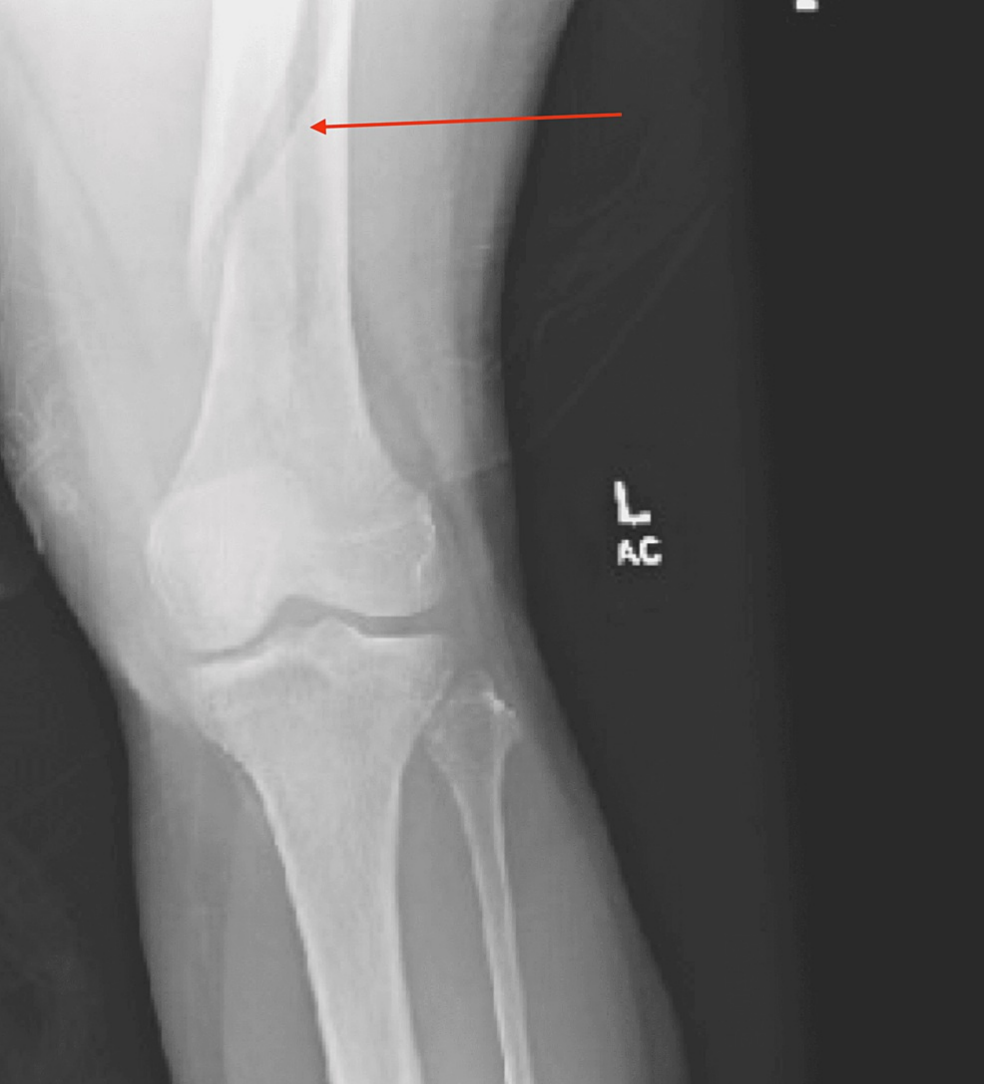
Radiograph of the left knee The image revealed a left closed, displaced, comminuted fracture of the left distal femoral shaft (arrow)

Radiographic imaging of the left knee revealed a left closed, displaced, comminuted fracture of the left distal femoral shaft (Figure 1). Orthopedic surgery was consulted and the patient was scheduled for open reduction internal fixation (ORIF) with intramedullary nail pending preoperative investigation with a chest X-ray (CXR) and electrocardiogram (EKG). CXR demonstrated no evidence of cardiopulmonary disease or acute pathology. EKG showed sinus rhythm, with a rate of 62 beats per minute, normal axis, normal PR interval, and no acute ischemic changes.

The patient's past medical history was significant for hypertension, anxiety, and chronic lower back pain. Airway and cardiopulmonary examinations were within normal limits. Her home medications included losartan, labetalol, and clonazepam. She was a former smoker, with a 15-pack-year smoking history and had quit 30 years prior to the current admission. The patient had no known drug allergies. Her surgical history included septoplasty, incision and drainage of a thigh abscess, dental implants, and colonoscopy, all without any anesthetic complications.

Preoperative regional anesthesia included a femoral nerve block with 30 ml of 0.5% ropivacaine. General anesthesia was induced with 100 mg of propofol, 25 mcg of fentanyl, and 70 mg of lidocaine, and maintained with sevoflurane. The airway was secured with a size 4 Unique laryngeal mask airway (LMA). The LMA was selected as the airway of choice as this was an elective case, the patient met appropriate nil per os (NPO) guidelines, and no paralysis was required for the procedure. The make was selected based on its ready availability at the facility, and sizing was appropriately selected based on patient weight, with size 4 LMA recommended for patients weighing 50-70 kg. Spontaneous ventilation via the LMA was maintained with a 100% fraction of inspired oxygen (FiO_2_), oxygen flows of 2 liters per minute, and sevoflurane at a minimum alveolar concentration (MAC) of 2.2% on GE Aisys Carestation anesthesia machine (GE HealthCare, Chicago, IL).

Approximately 180 minutes after the start of the procedure, and immediately following proximal interlocking screw placement, the patient’s oxygen saturation decreased from 100% to 91%. Concurrently, end-tidal CO_2_ values decreased from 36 to 28, while heart rate elevated by 5-7 beats per minute from a baseline of 75 beats per minute. The LMA was removed and replaced to confirm adequate positioning, and it was noted to be free of secretions and gastric contents. The patient's oxygen saturation failed to improve, prompting intubation with endotracheal tube size 7.0. She became hypotensive, with blood pressure ranges of 90/50 - 80/45 mmHg, requiring repeat doses of phenylephrine boluses to maintain a mean arterial pressure (MAP) of 65. The differential of FES versus PE was considered given the presence of respiratory distress, the surgical procedure, and clinical suspicion. 

Cardiovascular and hemodynamic stabilization were obtained following intubation. Three ephedrine boluses (20 mg, 10 mg, and 20 mg), six phenylephrine boluses (200 mcg, 200 mcg, 200 mcg, 200 mcg, 100 mcg, and 100 mcg), and three vasopressin boluses (4 units, 3 units, and 1 unit) respectively were administered intermittently upon the onset of symptoms for the initial management of possible shock state, utilizing vasoconstrictive properties in an attempt to improve blood flow and oxygen delivery to tissues. Upon completion of the procedure, the patient was transported to the ICU intubated.

**Figure 2:**
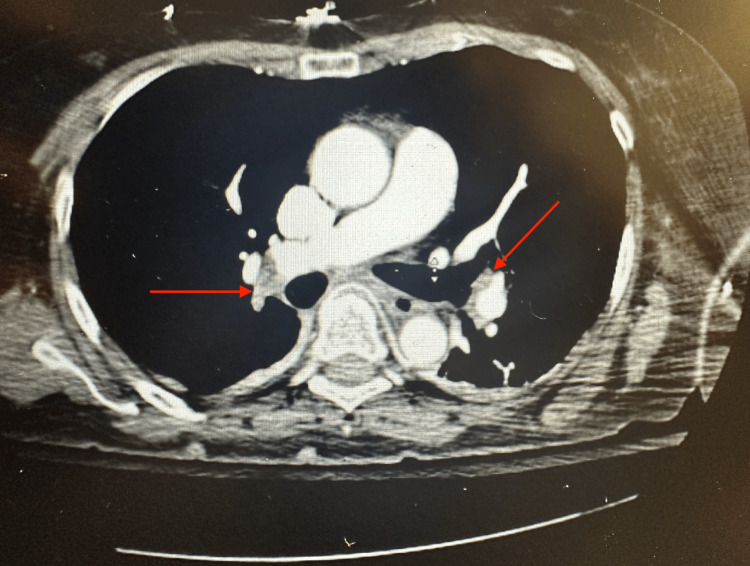
Postoperative CT angiogram of the chest The image demonstrated bilateral subsegmental pulmonary emboli (arrows) CT: computed tomography

In the ICU, multiple pale red petechiae were noted on the anterior and anterolateral thorax, which increased suspicion regarding a diagnosis of FES. A confirmatory CT angiogram of the chest was performed, which revealed acute bilateral subsegmental PE (Figure 2). There was no right ventricular strain, hemodynamic instability, or other acute abnormalities, which was confirmed via echocardiogram. The patient was not a candidate for thrombectomy, and tissue plasminogen activator (tPA) was not administered due to the patient having undergone a surgical procedure. A heparin drip was initiated in the ICU for the management of PE, at a dose of 80 units/kg and a target partial thromboplastin time (PTT) of 43 seconds or less.

The patient was extubated to a high-flow nasal cannula on postoperative day one. On postoperative day three, she was downgraded from the ICU and eventually discharged to an inpatient rehabilitation facility on postoperative day six.

## Discussion

FES is a rare complication associated with orthopedic procedures, such as ORIF of long bone fractures [1]. In FES, fat globules are released into the bloodstream, and the subsequent obstruction of blood vessels can result in respiratory, cardiovascular, and neurological complications [2]. The incidence of FES has been reported in 0.5-11% of patients who experience long bone fractures [3]. Among those, mortality has been reported in approximately 1-10% of cases, although some studies have reported that mortality rates can be as high as 20% [1-3]. The highest risk for morbidity and mortality is linked to the presence of severe respiratory or cardiovascular complications [1,3]. Common precipitants of severe cases of FES are delays in the identification and treatment of the symptoms [3].

The diagnosis of FES is clinical and usually based on a triad of respiratory distress, neurological dysfunction, and petechial rash with salmon-colored hues [1-4]. Major and minor diagnostic criteria for FES diagnosis were proposed by Gurd and Wilson, which are depicted in Table 1 [6-7]. According to these criteria, FES could be diagnosed based on the presence of one major feature, four minor features, and fat macroglobulinemia. Our patient was treated with a presumptive clinical diagnosis of FES due to high clinical suspicion, evidenced by the presence of decreased oxygen saturation and a characteristic petechial rash.

**Table 1:** Summary of major and minor diagnostic criteria for FES as proposed by Gurd and Wilson FES: fat embolism syndrome

Gurd and Wilson major and minor criteria for diagnosing FES	
Major criteria	Minor criteria
Petechial rash	Tachycardia
Respiratory symptoms with radiographic changes	Pyrexia
Cerebral signs unrelated to head injury	Retinal fat or petechiae
	Urinary fat globules
	Sudden drop in hemoglobin (Hb) level
	Sudden thrombocytopenia
	High erythrocyte sedimentation rate (ESR)
	Fat globules in sputum

Despite several years of extensive clinical research, standardized treatment guidelines for cases of FES have not been devised [3]. The treatment usually involves supportive measures, which focus on maintaining oxygenation and ventilation, providing hemodynamic support, and resuscitation with fluids and/or blood products, if required [8]. A high flow rate of oxygen and maintenance of intravascular volume are important to maintain arterial oxygen tension and prevent shock, which can worsen lung injury in FES [8]. Furthermore, mechanical ventilation and positive end-expiratory pressure (PEEP) may be necessary to ensure adequate arterial oxygenation [9]. Studies have reported that steroids, alcohol, dextran, and heparin have limited efficacy in FES management [10-11]. Heparin administration, while part of the standard management of PE, should be used with caution in the setting of FES, a pro-inflammatory state. Reports have demonstrated an increase in mortality, thought to occur secondary to a rise in free fatty acid content due to accelerated hydrolysis of fat emboli lodged in the lungs. Free fatty acids act as toxins within the lungs, leading to pneumocyte and pulmonary endothelial cell injury and damage. This further activates inflammatory processes and cytokine cascade, leading to acute lung injury or even acute respiratory distress syndrome.

However, some studies have demonstrated that corticosteroids may be beneficial when used as prophylaxis in preventing FES and hypoxia in patients with long-bone fractures [12-13]. The role of albumin as a means of resuscitating patients with FES who suffered traumatic brain injuries (TBI) remains controversial [14]. Cardiovascular stability and oxygenation in FES are commonly managed with one or a combination of fluids, peripheral vasoconstrictors, inotropic drugs, or pulmonary vasodilators [14]. In our patient, intubation, mechanical ventilation, fluid replacement, and vasopressor support were required to maintain a MAP of 65 mmHg and ensure cardiovascular and hemodynamic stability.

PE is another possible complication of operative procedures, in which a blood clot travels to the lungs and causes respiratory distress. While PE is associated with decreased oxygen saturation, its pathophysiology differs from that of fat embolism syndrome, which is mediated by the activation of the complement system and leukocyte aggregation [1-2]. Furthermore, isolated PE does not produce the classic petechial rash that is characteristic of FES. The treatment of PE typically involves anticoagulant therapy, such as heparin or warfarin, to prevent further clot formation and dissolve existing clots [5]. In a patient with FES who subsequently develops PE and has potential bleeding complications that could arise from administering anticoagulant therapy, the decision to administer anticoagulants needs to be carefully weighed against the potential benefits and risks. Our patient was not a candidate for thrombectomy, and tPA was not administered due to the patient having undergone a surgical procedure, which is a common contraindication for thrombolytic therapy, especially tPA [15]. Contraindications for thrombectomy in patients with PE include recent major surgery, ongoing or recent bleeding, neoplastic disease, traumatic injuries, suspected bleeding, or recurrent PE despite thrombolytic therapy [1]. Our patient, however, was considered stable enough for heparin management for PE, as the benefits outweighed the bleeding risks.

This case report highlights the importance of maintaining a high index of suspicion for FES and PE in patients undergoing orthopedic procedures. Rapid recognition and treatment of these complications can improve outcomes and prevent morbidity and mortality among patients. Further research is needed to identify risk factors and devise optimal treatment strategies for FES and PE in this patient population.

## Conclusions

We discussed a case involving the development of FES and multiple small pulmonary emboli following ORIF of a left femur fracture in a 56-year-old female patient who had suffered a mechanical fall. The patient was managed with supportive care, which included mechanical ventilation, fluid resuscitation, and vasopressor support. Additionally, heparin was administered to prevent further clot propagation. Although the management of FES varies on a case-by-case basis, depending on the degree of severity and setting in which it is identified, awareness about the possibility of this condition and maintaining a high index of suspicion during the intraoperative course are critical as prompt recognition and treatment can improve outcomes and prevent patient morbidity and mortality.
